# Supplementary material for: Is the whole the sum of its parts? Neural computation of consumer bundle valuation in humans
Source: bioRxiv. 2025 May 1:2025.04.28.650827. Preprint. [Version 1] doi: 10.1101/2025.04.28.650827 (PMC12247921; doi:10.1101/2025.04.28.650827)
Supplement: 1 [file NIHPP2025.04.28.650827V1-supplement-1.pdf]

bioRxiv preprint doi: https://doi.org/10.1101/2025.04.28.650827; this version posted May 1, 2025. The copyright holder for this preprint (which was not certified by peer review) is the author/funder, who has granted bioRxiv a license to display the preprint in perpetuity. It is made available under aCC-BY 4.0 International license.

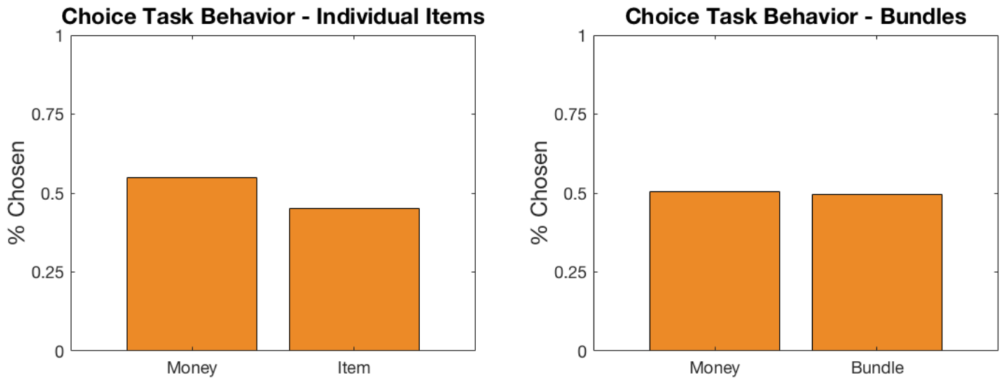

Figure 6: **Supplementary Figure 2. Behavior on the choice task.**
Percentage of trials in which the item or bundle was chosen vs. the reference monetary amount.

bioRxiv preprint doi: https://doi.org/10.1101/2025.04.28.650827; this version posted May 1, 2025. The copyright holder for this preprint (which was not certified by peer review) is the author/funder, who has granted bioRxiv a license to display the preprint in perpetuity. It is made available under aCC-BY 4.0 International license.

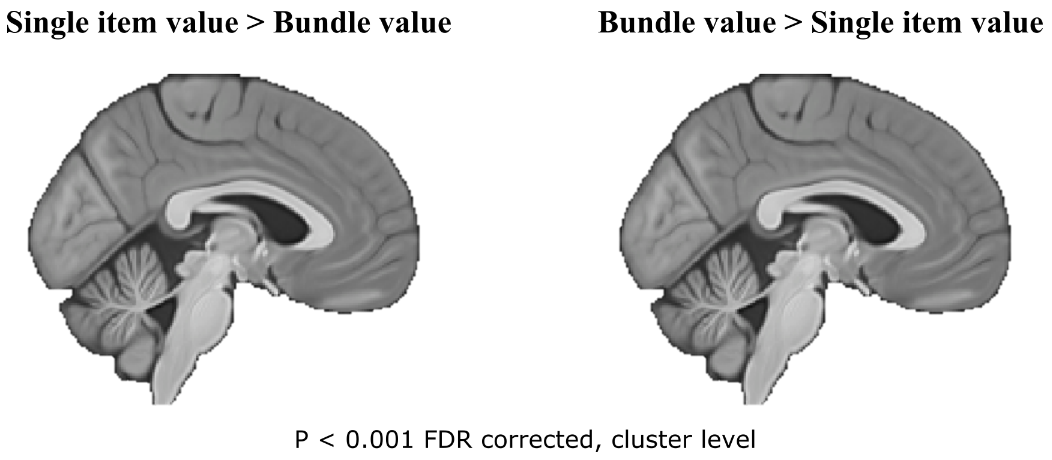

Figure 7: **Supplementary Figure 3. Bundle Value vs Single Item Value.**
Univariate contrasts testing the interaction of value and trial type. No clusters survived in either comparison after multiple comparisons correction.

bioRxiv preprint doi: https://doi.org/10.1101/2025.04.28.650827; this version posted May 1, 2025. The copyright holder for this preprint (which was not certified by peer review) is the author/funder, who has granted bioRxiv a license to display the preprint in perpetuity. It is made available under aCC-BY 4.0 International license.

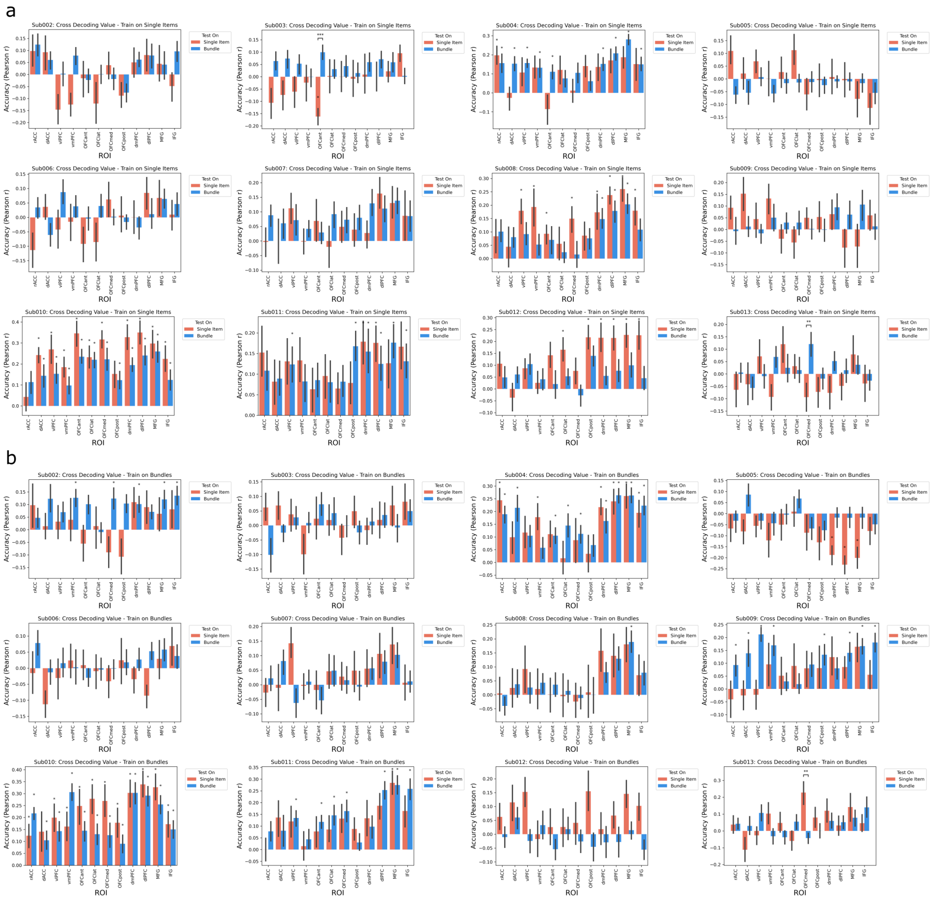

Figure 8: **Supplementary Figure 4. Bundle Value vs Single Item Value.**
MVPA cross decoding analysis individual subject results. Left: decoders trained on trials of single items. Right: decoders trained on bundle trials. Asterisks * represent significant prediction accuracies on a test partition (two-sided one-sample Wilcoxon signed rank test $P < 0.05$ and FDR-corrected for multiple comparisons $q = 0.05$). At the group level, there were no significant paired differences in prediction accuracies between test conditions for any ROI (two-sided two-sample Wilcoxon signed rank test $P < 0.05$ and FDR-corrected for multiple comparisons $q = 0.05$). Error bars reflect SE across participants.

bioRxiv preprint doi: https://doi.org/10.1101/2025.04.28.650827; this version posted May 1, 2025. The copyright holder for this preprint (which was not certified by peer review) is the author/funder, who has granted bioRxiv a license to display the preprint in perpetuity. It is made available under aCC-BY 4.0 International license.

Food items used	
1. 3 Musketeers	36. Sun Chips
2. Barnum's Animal Crackers	37. Dole Mixed Fruit
3. Doritos Nacho Cheese	38. Grapefruit
4. Chips Ahoy!	39. Banana Chips
5. Kit Kat	40. Dark Chocoloate Bananas
6. Pop-Tarts Brown Sugar Cinnamon	41. Crispy Apple
7. Pop-Tarts Brown Sugar Strawberry	42. Vegetable Chips
8. Ghiradelli Chocolates	43. Sweet Potato Chips
9. Twix Cookie Bars	44. Chopped Salad Chicken
10. Hershey's Whatchamacallit Candy	45. Mexicali Salad
11. Apple Pie	46. Caesar Salad
12. Avocado	47. Veggie Wrap
13. Blackberries	48. Super Burrito
14. Cauliflower	49. Chocolate and Berry
15. Ritz Crackers'n Cheese Dip	50. Green Beans Chips
16. Cherry Pie	51. Salami
17. Chocolate Muffins	52. Smoked Turkey
18. Powdered Donuts	53. American Cheese
19. Granny Smith Apple	54. Chicken and Roasted Beet
20. Green Grapes	55. Mozzarella Cheese
21. Mango	56. Roast Beef
22. Milano Cookies	57. Caprese Sandwich
23. Orange	58. Tuna Salad Wrap
24. Raspberries	59. Smoked Salmon
25. Red Velvet Cake	60. Plain Yogurt
26. Quaker Chewy Granola Bar	61. Strawberry Yogurt
27. Starburst	62. Blueberry Yogurt
28. Strawberry	63. Deviled Eggs
29. Crunchy Donuts	64. Smore's Chewy Bars
30. Chicken Tikka Masala	65. Gnocci
31. Lamb Vindaloo	66. Magherita Pizza
32. Pollo Asado Burrito	67. Macarons
33. Bean and Cheese Burrito	68. Blueberry Crisp Clif Bars
34. Chocolate Chip Clif Bars	69. Yogurt Pretzels
35. Ferrero Chocolates	70. Chocolate Pretzels
**Consumer goods used**	
1. A Brief History of Time book	21. Lock
2. Freakonomics book	22. Notebook
3. 1984 book	23. Bathroom scale
4. Water bottle	24. Playing cards
5. Wireless mouse	25. Honey clementine candle
6. Yoga mat	26. Roses candle
7. Hitchhikers book	27. Umbrella
8. Lord of the Rings book	28. Android charger
9. Caltech backpack	29. iPhone charger
10. Caltech hat	30. Clothes hangers
11. Caltech banner	31. Beach towel
12. Caltech keychain	32. Cooking supplies
13. USB stick 16GB	33. Kitchen utensils
14. Caltech mug	34. Pens
15. Caltech drawstring bag	35. Plates
16. Desk lamp	36. Portable charger
17. Stapler	37. Portable speaker
18. Over the ear headphones	38. Screwdrivers
19. Head backpack	39. Sunglasses
20. Batteries	40. Surge Protector

Table 1: Items used in experiment.
